# What does family building mean? A qualitative exploration and a new definition: a UK-based study

**DOI:** 10.1186/s12978-022-01511-w

**Published:** 2022-10-28

**Authors:** Bola Grace, Jill Shawe, Geraldine Barrett, Nafisat Ohunene Usman, Judith Stephenson

**Affiliations:** 1 Research Department of Reproductive Health, UCL EGA Institute for Women’s Health, Faculty of Population Health Sciences, University College London, Room 236 Medical School Building, 74 Huntley Street, London, WC1E 6A UK; 2 Faculty of Health, University of Plymouth Devon, Plymouth, UK; 3 SW Clinical School, Royal Cornwall Hospital, Truro, UK; 4 Department of Community Medicine, Kaduna State University, Kaduna, Nigeria

**Keywords:** Family planning, Family building, Fertility awareness, Psychosocial, Reproductive Health, Qualitative research, Childbearing, Preconception health, Reproductive intentions, Fertility education

## Abstract

**Background:**

The importance of improving men’s and women’s knowledge of sexual and reproductive health has been emphasised in numerous global health policies. Fertility awareness literature highlights a disproportionately higher number of articles related to pregnancy-prevention compared to pregnancy-planning, which is justifiable in many contexts. However, recent concerted effort to improve fertility-awareness warrants a closer investigation of basic reproductive health terminologies. The objective of this study is to explore participants’ views of “family building” and provide a definition.

**Methods:**

We conducted 35 qualitative in-depth interviews on men, women and healthcare professionals who were sampled from a UK cross-sectional survey. We asked participants about terms such as ‘family planning’ and ‘family building’ to elicit views and explored the appropriateness of the term “family building.” Data were transcribed and analysed via Framework analysis.

**Results:**

When asked what ‘family planning’ meant to them, study participants stated that the term meant the avoidance of pregnancy. They viewed it as an “*umbrella term for the use of contraception methods,*” that “*paradoxically, the term family planning almost has a negative connotation regarding having a family*,” but could not state similar terminology for planning a family. Reasons cited for this perspective include the focus of school education and usage in clinical settings.

**Conclusions:**

In the absence of an explicit definition in literature, we generated a new definition for family building as follows: “*Family building refers to the construction or formation of a family, which can include steps or actions taken by an individual towards having children. In contrast to family planning, the intent focuses on pregnancy planning and childbearing rather than pregnancy prevention. However, it can also include actions taken to space the number of children one has.*” Some balance in the global public health messages, including bridging the gap in reproductive health literature, policies, processes and practices may contribute to the effort to improve fertility knowledge. Use of appropriate terminologies help optimise reproductive health services in order to enable men and women achieve their desired fertility intentions, whatever they may be.

*Trial registration* Not applicable

## Plain language summary

Global health policies have emphasised the importance of improving individual’s knowledge of sexual and reproductive health. Fertility awareness literature highlights a disproportionately higher number of articles related to pregnancy-prevention compared to pregnancy-planning, which is justifiable in many contexts. However, the recent concerted effort to improve fertility awareness warrants a closer investigation of basic terminologies in the field. For example, although the term family planning encompasses attaining the desired number of children and spacing pregnancies, it is almost synonymous with not having children, while there is currently no widely accepted equivalent terminology for planning to have children, either in general usage or clinical settings. We conducted 35 qualitative in-depth interviews on men, women and healthcare professionals who were sampled from a UK cross-sectional survey. When asked what ‘family planning’ meant to them, study participants stated avoidance of pregnancy. They viewed it as an “*umbrella term for the use of contraception methods*”, that “paradoxically, the term family planning almost has a negative connotation regarding having a family,” but could not state similar terminology for planning a family. We introduced family building and provided a new definition. We believe that some balance in the global public health messages, including revisiting widely used terminologies can help bridge the gap in reproductive health literature, and contribute to the effort to improve fertility knowledge. Additionally, this has implications for promotion of preconception and optimising reproductive health in relevant policies, processes and practices, in order to help people achieve their desired fertility intentions, whatever they may be.

## Background

Global health policies have highlighted the importance of optimising women’s health and knowledge of contraception as part of pregnancy prevention and for pregnancy planning as part of preconception care [1–4]. However, in terms of general fertility awareness, there is far more information and education on pregnancy prevention than pregnancy planning. A vast number of studies have been conducted worldwide assessing the level of education on contraception for pregnancy prevention, however but not as many studies have been conducted for getting pregnant. In sexual and reproductive health (SRH) education, there is generally more emphasis on pregnancy prevention than pregnancy planning. This skew in fertility awareness is likely due to decades of global campaigns that have focussed on the reduction of abortion and unintended pregnancies [5, 6]. Since unintended pregnancy, especially in adolescence, continues to be a key contributor to adverse maternal and child outcomes [7] as well as perpetuating a cycle of poverty and poor health [8], decades of public health initiatives have emphasised the importance of expanding access to contraception [9]. These global campaigns are justified; studies have shown that a third to a half of all pregnancies across the world are unintended and around 80% of women affected do not use modern contraception [10]. Reviews of worldwide pregnancy trends [11, 12] showed that, after declining significantly between 1995 and 2008, the global rate of pregnancy decreased only slightly between 2008 and 2012. However, eighty-five million pregnancies (representing 40% of all pregnancies) were unintended in 2012. Of these unintended pregnancies, 50% resulted in abortion, 13% resulted in miscarriage and 38% ended in an unplanned birth. Between 2012 and 2014, 59% of unintended pregnancies in developed countries and 55% in developed countries resulted in abortion. These findings emphasise the importance of contraception.

Family planning can be linked back to falling birth rates from the late nineteenth century, differentially among the social classes, the demographic transition and the role, or otherwise, of individual rational action. Pre- and early-twentieth century artificial methods of contraception were still associated with non-marital sex and immorality with historians attributing most of the declining birth rate to natural methods [13]. By the beginning of the twentieth century, various drivers, such as concerns about population fitness and over-breeding of the lower classes, eugenics, feminism, cultural change and norms had an impact on family size. The change in status of contraception from morally unacceptable to state sanctioned happens through the 1920s, 30 s and beyond. Different group activists used different arguments, but the unifying approach was to dissociate contraception from sex or sexuality as much as possible and link it to other benefits, such as increasing population fitness, improving women's health, relieving poverty [14]. Therefore, arguments related to sex within marriage, by default related to procreation and family. Promoting the respectability, or non-sexual aspects of contraception was key. The National Birth Control Association, which was set up in the 1920s, changed its name to the Family Planning Association in 1939. Hawkes [15] and Weeks [16] both see this as significant, linking contraception to the growing social planning, intervention of governments at this time. Hawkes goes on to argue that "family planning" and the "prioritization of planned families" allowed the state to manage fears about troublesome non-marital sexuality and procreation.

Although the concept of family planning encompasses attaining one’s desired number of children and spacing pregnancies, it is typically attributed to the practice of limiting the number of children one has. In general usage, it is synonymous with birth control, use of contraception and prevention of unwanted pregnancy. According to Oxford dictionaries [17], family planning can be defined as “the practice of controlling the number of children one has and the intervals between their births, particularly by means of contraception or voluntary sterilisation”. A This shows a gap in literature with needing further exploration. This important emphasis on the prevention of unintended pregnancies, means that, by comparison, relatively little attention is paid to the decline in global fertility which is now below replacement level fertility in many developed countries [18]. Potential explanations for this decline include the postponement of childbirth [19] as well as involuntary childlessness [20].

While the use of the term family planning for pregnancy prevention is widely established in general literature as well as scientific and clinical settings, in conducting our literature review for this study, it was evident there was no equivalent widely used terminology for describing a focus on achieving pregnancy. “Proception” [21] has been used as a term to describe the behaviour in which the goal is to achieve conception but it has never moved into wider use. “Family-building” also appears in the literature [22–28] but no definition of this term is provided in literature and the origin in the context of fertility awareness is unknown; however, the term is used more frequently in the United States than elsewhere. There is also evidence that this term has been in used in research papers since the 1970’s [29], but explicit definition has not been provided. A consequence of a misunderstanding of family planning in the context of wanting more children means that there may be disparities in public health messaging on reproductive health. Although there is still an important need for continuous advocacy of contraception within fertility awareness campaigns, some balance in the message may be required to alleviate problems that people experience with family building, with the usage of fertility related terminologies being an important component. This study explores participants’ views of the terms “family planning” and “family building” in the context of a project about fertility awareness, reviews the appropriateness of the term “family building” and in the absence of an explicit definition in literature, proposes a new definition.

## Methods

The study is a qualitative component of a wider mixed methods study. Participants were sampled from a UK wide cross-sectional survey on fertility awareness, including men, women and healthcare professionals who had agreed to a follow-up interview. Participant demographic information was collected as part of the survey questionnaire. A new study invitation email was sent to recruit for the qualitative interviews. Criteria based purposive sampling was systematically employed to cover the socio-demographic diversity of the three population groups based on gender, age, ethnicity, and education, as well as to cover the range within each group. In this study, thirty-five interviews were conducted on thirteen men, thirteen women and nine healthcare professionals as previously described in Grace et al. [30]. Thirty-two remote interviews and three in-person face-to-face interviews were conducted by a single trained interviewer.

Interviews lasted one hour on average. As part of questions on their knowledge and attitudes towards fertility awareness, planned and unplanned pregnancies, participants were asked what the term “family planning” meant to them. The term “family building” was introduced to evaluate participants’ understanding. Our full definition was then introduced to study participants in order to elicit views:

“*Family building refers to the construction or formation of a family, which can include steps or actions taken by an individual towards having children. In contrast to family planning, the intent focuses on pregnancy planning and childbearing rather than pregnancy prevention. However, it can also include actions taken to space the number of children one has*”.

All interviews were digitally recorded, transcribed verbatim and coded electronically using the NVIVO Pro software, QSR International [31]. Data analysis was conducted using the Framework methodology, providing a structure into which the data can be systematically interrogated by the researcher, in order to analyse it by case and by code [32]. The coded framework matrix was exported from the NVIVO software into a Microsoft Excel file which was used for further examination, categorisation, and analysis. In summary, the data analysis process consisted of the coding of individual quotations verbatim, summarising quotations, grouping into higher order categories (themes), and conducting within theme analysis. In order to minimize personal bias, reflexive journaling was used, followed by a qualitative review workshop with five attendees (co-authors B.G., J.Sh. and J.St. and two qualitative research experts within the university department).

Ethical approval was obtained from UCL Research Ethics committee (Reference 8421/001). All participants in this study participated voluntarily and gave informed consent. The interviews were conducted sensitively by a single trained interviewer.

## Results

### Participant sociodemographic characteristics

The sociodemographic characteristics of study participants are shown in Table 1.

**Table 1:** Sociodemographic characteristics

Characteristic	Category		
Lay population group (n = 26)		Male (*n* = *13)*	Female *(n* = *13)*
Age group	18–27 years	3	4
	28–36 years	5	5
	37–45 years	5	4
Ethnicity	Asian	3	4
	Black	–	2
	White	10	5
	Mixed	–	2
Education	No degree	6	8
	Degree or equivalent and above	7	5
Healthcare professionals (*n* = *9)*		Male (*n* = *2)*	Female *(n* = *7)*
Age group	> 35	1	3
	< 35	1	4
Training	General practitioner (Primary care)	1	1
	Nurse (Primary care)	–	3
	Consultant (Secondary care)	1	1
	Doctor (Secondary care)	–	2

### Interview themes

#### Understanding—What does the term “family planning” mean to you?

When interview respondents were asked what they understood by the term family planning, by far the most common responses were that family planning meant contraception or pregnancy prevention.

*“Trying not have children”.* MP5—Male, Age 38, White, GCSEs, has two children, does not want more.

*“Family planning is just making sure you have knowledge about contraception really”* MP7—Male, Age 35, Chinese, Degree qualification, has no child, would like in future.

*“It is an umbrella term for the use of contraception methods.”* MP10—Male, Age 33, White, GCSEs, has no child, unsure about having children.

*“It’s obviously when you’re trying to plan so that you don’t end up having a baby that you don’t want to.”* FP5—Female, Age 30, Asian, A levels, has one child, would like more.

“*Family planning means using contraception*.” HCP4- Consultant, Male, Age 45, has three children, does not want more.

Interestingly, the majority of interviewees did not overthink this question. The view that the family planning terminology is used within the context of pregnancy prevention was simply an automatic response. However, some respondents were cognisant of the fact that together, the two words,’ family’ and ‘planning’, should mean planning a family which could include having children.

*“For me, family planning, I would think of it as trying to talk people out of having children before they’re ready and able to support them and bring them up in a stable and secure environment. Paradoxically, family planning for me almost exclusively has a negative connotation, and I don’t mean negative as in it shouldn’t happen. I mean negative as in it should be trying to help people plan properly for a family rather than prevention therefore holding them back rather than encouraging them to have a family.”* MP9—Male, Age 43, White, Degree qualification, has three children, does not want more.

For some respondents, the scope of the terminology goes beyond contraception and pregnancy prevention; it also covers preventing sexually transmitted infections, usage of screening services, abortion clinics and including other topics relating to sexual and reproductive health within clinical settings. Female respondents were more likely to consider the terminology in the context of women’s health generally.

*“So, family planning I think of women’s health, I think of screening, I think of contraception information and so it can be ranging from giving out the condom to young people to testing for STDs to kind of a place to go from the whole like it’s your health issue.”* FP11—Female, Age 21, Black, Degree qualification, has no child, would like in future.

*“I think of, like, doctors’ surgeries and that kind of thing, like, doctors asking that kind of question, like how you’re managing your fertility, in quite a medical sense rather than a more casual sense. I usually think contraception though.”* FP12—Female, Age 21, White, Degree qualification, has no child, unsure about having children in future.

*“I think of family planning clinic. Offering contraception, sexually transmitted disease advice things like that.”* HCP3, Consultant, White, no child, did not meet partner until later in life.

#### Why does contraception or pregnancy prevention come to your mind when thinking about the term family planning?

Following on from the responses on what family planning meant, we probed further to understand the reason why family planning was viewed almost exclusively in terms of contraception. Most respondents were unable to clarify the reason why family planning was viewed in the context of pregnancy prevention but there were some suggestions, especially amongst healthcare professionals, that it is related to the use of the term within clinical contexts. Another key theme which emerged as a reason for this connection was the focus of sexual and reproductive health school education being primarily on pregnancy prevention.

##### Usage in clinical settings

One of the key reasons provided for viewing family planning in terms of pregnancy prevention is the way it is used in clinical settings. For example, ‘family planning clinics’ are healthcare centres where patients would go to receive contraceptive services. These include clinics where patients are provided with condoms, placed on the pill, have contraceptive devices fitted or where abortion services are provided.

*“Contraception that’s the first thing that comes to mind. I have [a] medical background. I think always in terms of medicine that you know Family Planning Clinic is where people go to get their contraception or to have coils changed or to be started on the pill, after their pill maintenance, you know, having their blood pressure checked and for prescriptions.”* HCP1—Doctor, Female, Age 33, has two children, does not want more.

*“I think of a Family planning clinic. Offering contraception, sexually transmitted disease advice things like that. Americans have Planned Parenthood, which I get the impression is the same kind of thing. It’s just the general culture of talk about family planning is that it’s about [pregnancy] prevention”* HCP3—General Practitioner, Female, Age 45, has no child, would like in future.

##### Focus of school education

There were also ubiquitous themes regarding school education and the focus on pregnancy prevention, when family planning discussions came up as part of sexual and reproductive health education.

*“I remember at school it was all about not having a baby—there was nothing on planning for the future. I know you don’t want to have teenage pregnancies but when it comes to wanting to have a baby, I think it gives you a false understanding of how easy it is to have a baby because for some people it really isn’t. So, it’s really about understanding whole fertility and contraception at an earlier stage I think”.* FP2—Female, Age 32, White, Degree qualification, has one child, would like more.

*“We didn’t learn a lot on fertility as a whole. It was based on the fear factor. We were told not to get pregnant, not to have sex. Basically, avoid pregnancy at all costs.”* HCP2—Nurse, Female, Age 30, has no child, unsure about having children.

*“Thinking of family planning terminologies in school, there was quite a lot about contraception.”* HCP3—General Practitioner, Female, Age 45, has no child, would like in future.

*“I don’t think that we got any good foundation on fertility, the knowledge was far more sketchy and scant.”* HCP4- Consultant, Male, Age 45, has three children, does not want more.

#### Introducing “family building”—what does this term mean to you?

##### Planning for having children in the future

When the term “family building” was introduced to respondents, they were able to view the terminology in the context of wanting to have children.

*I think it [family building] involves looking to the future. Creating a family within a relationship.* MP5, male, Age 38, white, vocational qualification, has two children, does not want more.

*When I think of family building, I think of sitting down, having a talk with your significant other and just thinking about having a baby or furthering the relationship that way, that’s what I think*. MP2, male, Age 27, Asian, Degree, no children, would like in future.

##### Representation

Some respondents provided further reflection on the general perception of what family planning means, welcomed the new definition and reiterated the importance of representing needs for those wanting pregnancy prevention as well as pregnancy planning.

*“Honestly, I’m now not sure why I usually think of contraception. It [family planning] Should be about planning your whole reproduction, it’s not just about contraception. Family building makes me think of that. Yes, the whole discussion should be holistic to include having and taking a break from having children.”* FP1. Female, Age 36, Asian, Degree and above, has two children, would like more.

*“Yeah [family building] I’d think trying to have a baby or children in future. I guess that's what family planning ought to mean if you look at it holistically.”* HCP2—Nurse, Female, Age 30, has no child, unsure about having children.

“*…Your definition makes perfect sense. I guess family building is what we’ve missed from family planning.”* HCP3—General Practitioner, Female, Age 45, has no child, would like in future.

#### How do you think use of these terms can be improved to help people achieve desired fertility intentions?

Finally, interview respondents were asked to provide their views on improvement opportunities.

##### Inclusive language to frame reproductive health needs

In their responses, participants expressed the need for inclusive language to help patients better frame reproductive health needs.

*“So, I’d been on the pill for a long time before I decided that I wanted to have a baby, so I knew that my cycles weren’t really real. I do remember being worried about whether I’d be able to get pregnant or how long it would take. I was a bit unsure of going to the family planning clinic because I felt that they only deal with [contraception] pills… I feel they didn’t have the words or language to let women know about wanting babies.” FP5—Female, Age 30, has one child, would like more.*

##### Recognition of different fertility intentions

In recognition of different fertility intentions, there were views on balanced discussions on pregnancy prevention, pregnancy planning and reproductive life planning.

“*Yes, it should be a combination of both: talk about contraception and talk about planning for a baby. In discussions between couples, one person in the marriage or the relationship might want to start a family and then the other doesn’t. Having that discussion as to what's important to both parties and there should be a forum for that.”* HCP8—General Practitioner, Female, Age 36, has no child, would like in future.

##### Support tailored, age-appropriate and improved education

Linked to the perspective that school education mainly focussed on pregnancy prevention, there were recurring themes regarding improving school education, planting ‘the seed’ young in terms of early but age-appropriate fertility education.

*“It’s best to give the education [so] that they [students] can make the choices they want, rather than say ‘I wish I knew that 10 years ago’.”* FP11—Female, Age 21, Degree qualification, has no child, would like in future.

*“I think you should plant the seed when someone’s younger and then later on it should be there for them to get at the time when they want it, ‘cause everyone’s gonna want it at different times, aren’t they?”* HCP5—Nurse, Female, Age 24, has no child, would like in future.

## Discussion

This study aimed to explore participants’ views of the terms “family planning” and “family building” in the context of fertility awareness, review the appropriateness of the term “family building”, and in the absence of an explicit definition in literature, offers a new one. In line with literature findings, when asked what family planning meant to them, our study participants almost unanimously and automatically said prevention of pregnancy, which was a ubiquitous theme in this study. By contrast, they did not automatically offer a term for planning to have a family. One respondent highlighted that, “paradoxically, the term family planning almost has a negative connotation regarding having a family.”

To the best of our knowledge, this is the first study exploring the meaning of the terms “family planning” and “family building” among men and women of reproductive age as well as healthcare professionals. We believe the use of these terms in literature as well as scientific and clinical settings is an important component for improving fertility and reproductive health awareness to enable men and women achieve their desired fertility intention as its intent focuses on pregnancy planning and childbearing rather than pregnancy prevention, which family planning represents.

Due to the increased incidence of delayed family building, rates of involuntary childlessness and fewer children than desired have also increased [20, 26, 33]. Major cultural and sociodemographic changes, increased participation of women in the workforce and greater availability of contraception have all contributed to the ability to delay family building. Whilst there remains an important need for advocacy to improve access to contraception as part of family planning, this ought to be balanced with education on fertility, preconception care, healthy pregnancy and other factors involved in family building.

More collaborative initiatives from the different reproductive health services are needed. As highlighted by Shawe et al., “Proactive discussion of preconception care and pregnancy planning should be an integral part of all contraception and reproductive health services wherever they are provided”. In addition, new pregnancy-friendly terminologies are necessary within family planning services to encourage more balanced messages encompassing planning to have a family [34].

It is important to note that there are considerable variations in family building intentions among different individuals [35]. For those who desire children, improved education on fertility and factors that affect fertility for family building is crucial, but this should be provided within a societal context; and in recognition of today’s world, whereby socioeconomic and personal factors dominate choice over family building, and the harsh realities of biological constraints tend to be overlooked.

In order to address different and evolving needs, education and information packages on family building, ought to be tailored to be effective, however they are delivered. Services should be better equipped to educate both men and women about fertility awareness when they first report trouble conceiving to their HCP. This opens a potential role for general practitioners, practice nurses in primary healthcare services and sexual health practitioners, that is those working in sexual health and contraception services.

Information should be targeted based on different family planning and family building intentions using a lifecourse approach such as the Reproductive Life Plan (RLP), a set of personal goals regarding whether, when, and how to have children based on individual priorities, resources, and values [36]. This strategy would be a useful tool to implement. The RLP has been strongly advocated by many reproductive healthcare organisations and interest groups [36–40]. A research team in Sweden developed a protocol based on the RLP that aimed to encourage both women and men to reflect on their reproductive intentions as well as to find strategies for successful family planning, including achieving their desired fertility intentions and avoiding issues that could affect their reproductive health [37].

To encourage the engagement of men, the current female-focused awareness and education programmes on fertility and reproductive health generally should be reviewed and reoriented to be fully gender inclusive [30], creating an opportunity for inclusive innovation approaches. Additionally, educational programmes on sexual and reproductive health covering family building should be integrated within existing sexual and reproductive curricula ensuring there is a balance in reproductive health messages to include family planning and family building, including use of terms which cover the potential desire towards having child(ren) in future.

In terms of theoretic and practical application, beyond the use of the new definition in literature, it is important to consider how the use of terminologies could be promoted to help achieve desired fertility intention. We believe that proactive discussion of family planning and family building needs should be an integral part of reproductive health services [34, 41]. From our study, lay people and healthcare professionals felt that paradoxically, the term ‘family planning’ appears to have a negative connotation regarding having a family as the term is commonly linked to use of contraception. However, family planning clinics, known as Contraception & Sexual Health Clinics in the UK, tend to the first port-of-call for reproductive health needs. In embracing inclusive language through its use in literature, policy, guideline or by reproductive health organisations, the terminology helps frame patients' reproductive health needs more precisely and accurately. As such, we believe that the introduction of family building better describes the desires of those who are trying to have children rather than avoid children, helping them to achieve their desired fertility intentions.

In terms of study strengths and limitations, a key strength of this study is the original contribution to literature by providing a new definition for family building in the absence of an explicit definition. Another strength is the inclusion of men in the study as they are often left out of the pregnancy and childbearing discourse. In terms of study limitations, although we gathered rich data, interviewees were self-selected and results principally reflect views of those who we willing to participate. Due to the online recruitment method, there is a potential bias towards more educated respondents. Finally, family planning clinics in different countries across the world may serve different purposes, which has implications for the generalisability. While in principle, the study findings are applicable in similar contexts, the representativeness of the UK population would need to be considered.

## Conclusions

Our study provides as new definition for family building as follows: “Family building refers to the construction or formation of a family, which can include steps or actions taken by an individual towards having children. In contrast to family planning, the intent focuses on pregnancy planning and childbearing rather than pregnancy prevention. However, it can also include actions taken to space the number of children one has.” Historical global emphasis on pregnancy prevention means that, by comparison relatively little attention is being drawn to issues associated with postponement of childbirth and infertility. Although there is still an important need for continuous advocacy of contraceptive methods for family planning within fertility awareness campaigns, some balance in the global public health message may help alleviate problems experienced with involuntary infertility, including use of terminologies. Effective discussion of family planning and family building needs is integral to reproductive health services. This new definition creates an opportunity for those who are actively seeking to create or build a family to better express their needs and desires and engage constructively with healthcare professionals. It also has implications for inclusive language in the promotion of preconception and for optimising reproductive health in relevant policies, processes and practices, in order to enable men and women achieve their desired fertility intentions, whatever they may be.

## Data Availability

The datasets used and/or analysed during the current study are available from the corresponding author on reasonable request.
